# Normobaric oxygen treatment for mild-to-moderate depression: a randomized, double-blind, proof-of-concept trial

**DOI:** 10.1038/s41598-021-98245-9

**Published:** 2021-09-23

**Authors:** Yehudit Bloch, R. H. Belmaker, Pesach Shvartzman, Pnina Romem, Arkady Bolotin, Yuly Bersudsky, Abed N. Azab

**Affiliations:** 1 Department of Nursing, Faculty of Health Sciences, School for Community Health Professions, Ben-Gurion University of the Negev, P.O.B 653, 8410501 Beer-Sheva, Israel; 2 Department of Clinical Biochemistry and Pharmacology, Faculty of Health Sciences, Ben-Gurion University of the Negev, Beer-Sheva, Israel; 3 Southern District Health Office, Ministry of Health, Beer-Sheva, Israel; 4 Faculty of Health Sciences, Ben-Gurion University of the Negev, Beer-Sheva, Israel; 5 Department of Family Medicine and Siaal Center for Community Research, The Haim Doron Division of Community Health, Faculty of Health Sciences, Kappy and Eric Flanders National Palliative Care Resource Center, Ben-Gurion University of the Negev, Beer-Sheva, Israel; 6 Pain and Palliative Care Unit, Soroka Medical Center and Clalit Health Services, Beer-Sheva, Israel; 7 Department of Public Health, Faculty of Health Sciences, Ben-Gurion University of the Negev, Beer-Sheva, Israel; 8 Psychiatry Research Unit, Faculty of Health Sciences, Ben-Gurion University of the Negev and Mental Health Center, Beer-Sheva, Israel

**Keywords:** Depression, Randomized controlled trials

## Abstract

Oxygen enriched air may increase oxygen pressure in brain tissue and have biochemical effects even in subjects without lung disease. Consistently, several studies demonstrated that normobaric oxygen treatment has clinical benefits in some neurological conditions. This study examined the efficacy of normobaric oxygen treatment in subjects with depression. In a randomized, double-blind trial, 55 participants aged 18–65 years with mild to moderate depression (had a Hamilton Rating Scale for Depression [HRSD] score of ≥ 8) were recruited to the study from the Southern district in Israel. Participants underwent a psychiatric inclusion assessment at baseline and then were randomly assigned to either normobaric oxygen treatment of 35% fraction of inspired oxygen or 21% fraction of inspired oxygen (room air) through a nasal tube, for 4 weeks, during the night. Evaluations were performed at baseline, 2 and 4 weeks after commencement of study interventions, using the following tools: HRSD; Clinical Global Impression (CGI) questionnaire; World Health Organization-5 questionnaire for the estimation of Quality of Life (WHO-5-QOL); Sense of Coherence (SOC) 13-item questionnaire; and, Sheehan Disability Scale (SDS). A multivariate regression analysis showed that the mean ± standard deviation [SD] changes in the HRSD scores from baseline to week four were − 4.2 ± 0.3 points in the oxygen-treated group and − 0.7 ± 0.6 in the control group, for a between-group difference of 3.5 points (95% confidence interval [CI] − 5.95 to − 1.0; P = 0.007). Similarly, at week four there was a between-group difference of 0.71 points in the CGI score (95% CI − 1.00 to − 0.29; P = 0.001). On the other hand, the analysis revealed that there were no significant differences in WHO-5-QOL, SOC-13 or SDS scores between the groups. This study showed a significant beneficial effect of oxygen treatment on some symptoms of depression.

**Trial registration**: NCT02149563 (29/05/2014).

## Introduction

Depression is a devastating mental illness causing immense suffering among affected subjects and their families and is associated with severe emotional, functional and financial burden^1,2^. Depression is a common disorder especially among women^3,4^, with a life-time prevalence ranging between 10 and 20% in the general population^3,5^. These estimations may be misleading, the actual numbers presumably even higher, recognizing the cultural-associated differences in inferring and diagnosing mental illness^3,6^. The most widely used treatment strategies for depression are pharmacotherapy, electroconvulsive therapy and cognitive behavioral therapy^1,2,7–10^. However, despite the availability of therapeutic approaches, a high percentage of subjects with depression respond poorly, or not at all to administered treatments, with considerable amounts of them suffering unwanted side effects^1,2,11^. Importantly, few novel therapeutic interventions have been developed and introduced to clinical practice for the treatment of depression in recent years^12–14^. These data reinforce the need for a relentless search for novel efficacious and safe treatment options for depression.

Abnormal cellular energy metabolism due to functional deficiency in oxygen supply and/or mitochondrial dysfunction may lead to alterations in neuronal function, plasticity and brain circuitry. In a comprehensive review, Shao et al*.*^15^ summarized the evidence for the involvement of cellular energy metabolism abnormalities and mitochondrial dysfunction in psychiatric disorders and suggested that new treatment approaches should be directed to this research endeavor. Particularly, several studies reported mitochondrial function abnormalities in subjects with depression^16–18^.

Transfer of oxygen to the tissues in the human body depends almost entirely on the level and function of hemoglobin in the blood. Since almost all oxygen in the blood is bound to the hemoglobin molecule, only a tiny fraction is dissolved in plasma. Therefore, increasing the oxygen content in the air (at *normobaric* conditions) does not significantly increase the amount of oxygen carried to tissues, except in patients with respiratory disorders. These elements of basic physiology have discouraged studies on increasing the fraction of inspired oxygen under normal pressure in depression, as well as other neurological disorders. However, some studies suggested that the partial oxygen pressure (PO_2_) in the plasma of brain capillaries may be a physiological parameter that is not necessarily related to the oxygen carrying capacity in the blood (by hemoglobin)^19–21^. For instance, Menzel et al*.*^19^ reported that normobaric hyperoxia treatment (increasing the percentage of oxygen in inspired air *above* 21%) increased oxygen partial pressure in brain tissue, leading to improved mitochondrial function. More likely, the mechanisms of normobaric hyperoxia are complex, affecting various genes at a time and in a dose-dependent manner^22^. It is hypothesized that raising the pressure of the dissolved oxygen portion of the plasma affects oxygen pressure at key enzymes, perhaps in the mitochondria.

Normobaric oxygen treatment at a concentration of 40% was found safe in animal and human studies without evidence of pulmonary oxygen toxicity or neurological abnormalities^23^. On the other hand, the use of maximally high inspired oxygen percentage (100% O_2_) was found to reduce cerebral blood flow^24^. Studies using normobaric hyperoxia as treatment for some neuropathological conditions^25–27^ have shown positive results. Our previous study in patients with schizophrenia^28^ had encouraged us to conduct a similar study in patients with depression. Thus, the objective of the present study was to examine the effect of normobaric oxygen treatment (35% O_2_) on patients with depression. The fraction of inspired oxygen (FiO_2_), 35%, was decided based on the positive therapeutic effects observed in our study in patients with schizophrenia^28^ and the results of human studies demonstrating that a FiO_2_ in the range between ~ 30–70% evinced positive safety outcomes^28–30^.

In contrast to normobaric oxygen treatment, *hyperbaric* oxygen treatment may entail significant dangers^31–34^, and is possible only for short albeit repeated exposures. The definition of hyperbaric oxygen treatment is the administration of 100% oxygen under *increased* atmospheric pressure. This intervention can definitively increase the oxygen carrying capacity of blood to the brain. Efficacious use was reported in transient cerebral ischemia and in multiple sclerosis^35,36^. Efrati et al*.*^37^ found that hyperbaric oxygen treatment could activate neuroplasticity in patients with chronic neurologic deficiencies due to stroke. Hyperbaric oxygen treatment was also reported to improve post-concussion syndrome years after mild traumatic brain injury^38^. However, a major limitation of hyperbaric oxygen treatment is its high cost—it requires a special chamber which is expensive and available only in select major medical centers around the world. The treatment process is of approximately an hour duration requiring patients to travel to a specific, often remote, location. Furthermore, it can have severe side effects if the pressure rises too quickly or decreases too rapidly. Therefore, given the preceding obstacles surrounding hyperbaric oxygen treatment, we hypothesized the possibility that normobaric oxygen treatment may serve as a useful and attractive treatment strategy for depression.

## Results

**Figure 1:**
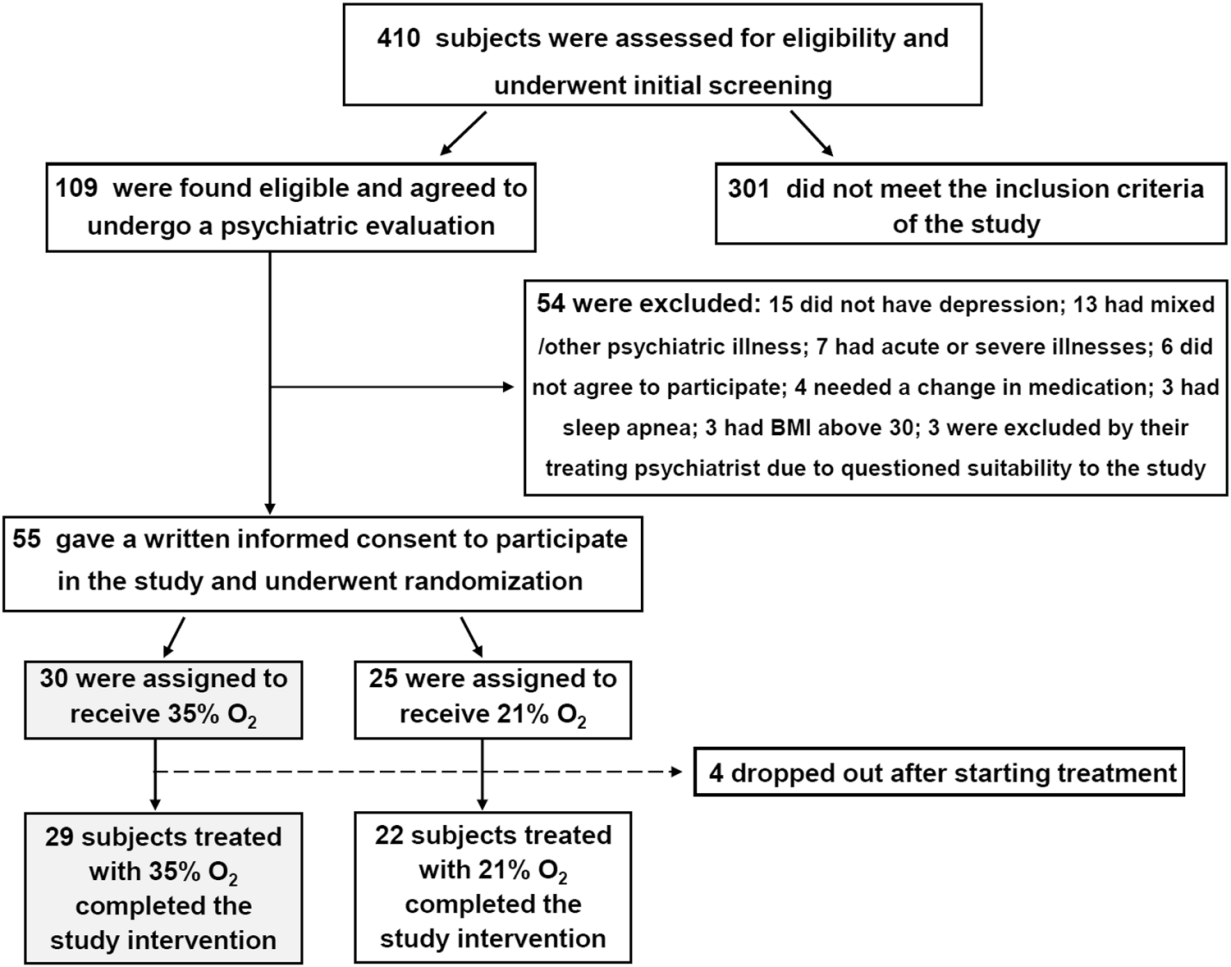
Screening and enrollment to the study. BMI denotes body mass index.

Fifty five subjects with mild to moderate depression were recruited between 2014 and 2019 and randomized to receive treatment with oxygen-enriched air (35% O_2_) for 1 month or room air (21% O_2_) for 1 month. Medications were not changed in patients who were enrolled to the study. Demographic and clinical characteristics of participants are summarized in Table 1. Figure 1 illustrates the flow diagram of recruitment, acceptance and assignment to the study groups. Fifty-one participants completed the study interventions and were included in the final analysis. Twenty-nine received oxygen-enriched air and twenty-two received room air. Four participants who completed less than 2 weeks (one—oxygen-treated, three—air-treated) were not included in the analysis.

**Table 1:** Demographic and clinical characteristics of recruited participants at baseline.

Characteristic^a^	Mean (SD)	
	O_2_ 35% (n = 30)	O_2_ 21% (n = 25)
Age, year	47.4 (11.2)	43.9 (13.5)
Age onset depression, year	35.83 (13.63)	33.24 (12.20)
Duration of illness, year	(9.85) 11.63	(7.45) 10.44
Body mass index	25.39 (3.84)	24.50 (3.27)
**Baseline score**		
HRSD	14.7 (3.9)	14.5 (3.1)
CGI-S	3.37 (0.49)	3.3 (0.46)
WHO-5-QOL score	6.89 (4.35)	6.87 (4.66)
SDS score	20.1 (7.0)	20.6 (6.2)
Arterial hemoglobin-oxygen saturation, %	96.95 (0.84)	97.18 (0.52)
	N (%)^b^	
**Depression severity at baseline**^c^		
Mild depression	18 (60.0)	18 (72.0)
Moderate depression	12 (40.0)	7 (28.0)
**Gender**		
Female	17 (56.6)	14 (56.0)
Male	13 (43.3)	11 (44.0)
**Employment status**		
Employed/partially employed	20 (66.7)	13 (52.0)
Unemployed	8 (26.7)	9 (36.0)
Pensioned	1 (3.3)	0 (0.0)
Student	1 (3.3)	3 (12.0)
**Marital status**		
Married	19 (63.3)	10 (40.0)
Divorced	8 (26.7)	6 (24.0)
Single	2 (6.7)	8 (32.0)
Widowed	1 (3.3)	1 (4.0)
**Educational level**		
High school	10 (33.3)	14 (56.0)
College or university	20 (66.7)	11 (44.0)
**Ethnic geographic origin**		
Ashkenazic	15 (50.0)	9 (36.0)
Sephardic	14 (46.7)	15 (60.0)
Arab	1 (3.3)	0 (0)
Other	0 (0)	1 (4.0)
**Cigarette smoking**		
No	15 (50.0)	16 (64.0)
Yes	15 (50.0)	9 (36.0)
**Underlying physical disorders**^d^		
No	15 (50.0)	8 (32.0)
Yes	15 (50.0)	17 (68.0)
**Family history of psychiatric illness**^e^		
No	18 (60.0)	16 (64.0)
Yes	15 (40.0)	9 (36.0)
**Previous use of antidepressant medication**		
No	9 (30.0)	6 (24.0)
Yes	21 (70.0)	19 (76.0)
**Antidepressant medication use at recruitment**^**f**^		
No	11 (36.7)	11 (44.0)
Yes	19 (63.3)	14 (56.0)

*CGI-S* Clinical Global Impression-severity, *HRSD* Hamilton rating scale for depression, *SD* standard deviation, *SDS* Sheehan disability scale, *SOC* sense of coherence, *WHO-5-QOL* world health organization-5 quality of life (questionnaire).

^a^Using *T*-test or chi-square test (according to type of parameter), there were no significant differences between the groups in any of the tested parameters.

^b^Percent in 35% O_2_-treated or 21% O_2_-treated groups.

^c^Depression severity based on Clinical Global Impression: mild—2–3, moderate—4.

^d^Including the following diseases: Attention deficit hyperactive disorder, fibromyalgia, hypothyroidism, cancer, anemia, eye disorder, heart disease, psoriasis, stomach/peptic disease, migraine, irritable bowel disease, other.

^e^Including the following illnesses: Anxiety disorders, bipolar disorder, depressive disorder, mental retardation, obsessive–compulsive disorder, posttraumatic stress disorder, schizophrenia, other.

^f^There was no difference between the groups in the incidence of a particular drug or a medication family (such tricyclic antidepressants or selective serotonin reuptake inhibitors).

### Effects of oxygen treatment on Hamilton Rating Scale for Depression (HRSD) score

**Figure 2:**
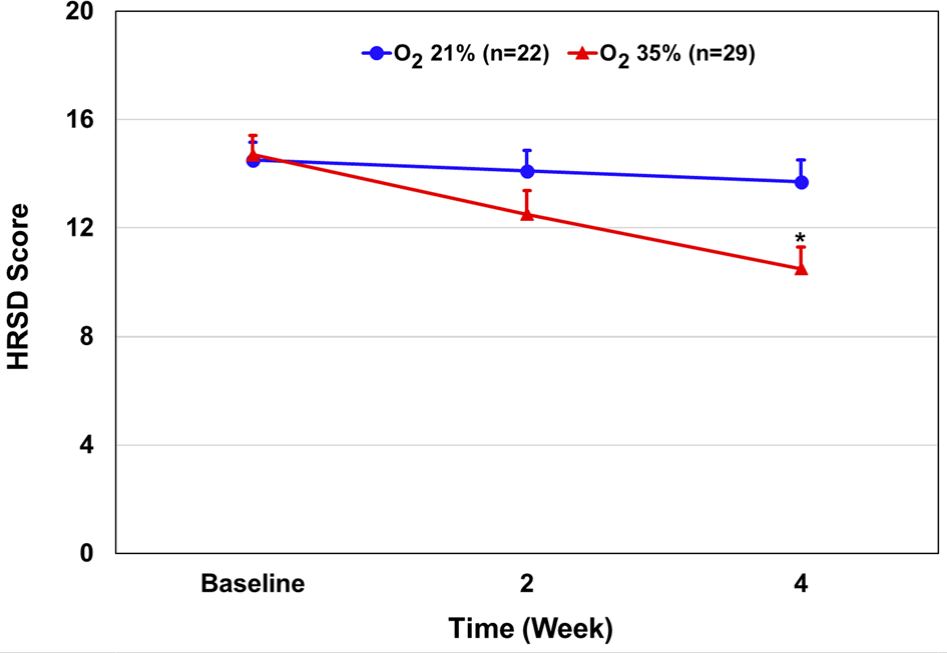
Effect of 35% oxygen treatment on total HRSD score. Results are presented as mean ± SEM. *Multivariate regression analysis: The mean ± SD changes in the scores from baseline to week four were − 4.2 ± 0.3 points in the oxygen-treated group and − 0.7 ± 0.6 in the control group, for a between-group difference of 3.5 points (95% confidence interval [CI] − 5.95 to − 1.0; P = 0.007).

Table 2 and Fig. 2 show the effect of oxygen treatment versus room air (control) on HRSD score in 51 patients with mild to moderate depression who completed the 1-month study. Oxygen treatment led to a significant reduction in HRSD total score compared with the control group (mean ± standard deviation [SD], 10.5 ± 4.2 vs. 13.8 ± 3.7) (Table 2). The mean ± SD changes in the scores from baseline to week four were − 4.2 ± 0.3 points in the oxygen-treated group and − 0.7 ± 0.6 in the control group, for a between-group difference of 3.5 points (95% confidence interval [CI] − 5.95 to − 1.0; P = 0.007) (Fig. 2). The point estimate of the mean treatment effect on HRSD score was 2.745, 95% CI [1.167, 4.323]. We tested the influence of the following variables: age, gender, marital status, education levels, adherence to treatment, and age of onset of depressive illness—on the effect of oxygen treatment on HRSD score. Only age of onset of depression had a significant effect (F (12, 42) = 3.95, Prob > F = 0.0004); a younger age of onset was associated with a more severe depression (higher HRSD score). However, the beneficial therapeutic effect of oxygen treatment on HRSD score remained significant even after correction for this variable. Importantly, age of onset of depression did not differ significantly between the groups at baseline (Table 1). Other variables such as cigarette smoking status, body mass index, depressive illness duration, and use and type of antidepressant medications at recruitment did not have a significant effect on the results.

**Table 2:** Effect of oxygen treatment on study outcomes.

Measure	Treatment	Baseline (mean ± SD)	Week 2 (mean ± SD)	Week 4 (mean ± SD)
HRSD	35% O_2_ (n = 29)	14.7 ± 3.9	12.5 ± 4.7	10.5 ± 4.2
	21% O_2_ (n = 22)	14.5 ± 3.1	14.1 ± 3.6	13.8 ± 3.7
CGI-I	35% O_2_ (n = 29)		3.4 ± 0.68	3.13 ± 0.74
	21% O_2_ (n = 22)		3.7 ± 0.53	3.77 ± 0.43
WHO-5-QOL	35% O_2_ (n = 29)	6.89 ± 4.35	9 ± 5.2	11.4 ± 4.85
	21% O_2_ (n = 22)	6.87 ± 4.66	8.4 ± 6.2	9.5 ± 6.097
SOC-13	35% O_2_ (n = 29)	50.13 ± 12.33	53.13 ± 13.7	57.3 ± 9.16
	21% O_2_ (n = 22)	46.8 ± 8.79	50.5 ± 8.2	53.0 ± 11.73
SDS	35% O_2_ (n = 29)	20.1 ± 7.0	16.8 ± 7.0	14.4 ± 7.6
	21% O_2_ (n = 22)	20.6 ± 6.2	19.9 ± 7.0	19.0 ± 8.0

*CGI-I* clinical global impression-improvement, *CI* confidence interval, *HRSD* Hamilton rating scale for depression, *SD* standard deviation, *SDS* Sheehan disability scale, *SOC* sense of coherence, *WHO-5-QOL* world health organization-5 quality of life (questionnaire).

Table 3 shows the multivariate regression analysis for the HRSD sub-scale factors. The reduction in the overall score of HRSD due to oxygen treatment was significantly due to the positive effect on *factor 1 − *anxiety/somatization (95% CI − 2.24 to − 0.51; P = 0.003), and *factor 3*—cognitive disturbance (95% CI − 1.66 to − 0.28; P = 0.007). Together, these factors led to nearly 53% of the total change in HRSD score; *factor 1* accounted for ~ 29% of the total reduction in HRSD and *factor 3* accounted for ~ 24% of the total reduction in HRSD. The change in the other *factors* was not statistically significant (Table 3). However, all the other items except diurnal variation were nominally more improved by oxygen treatment than room air. Many studies suggested that remission should be defined with a HRSD cut-off of seven or less^39–41^. Using that criterion, we found seven patients who reached remission with oxygen treatment and no patients in the control group who reached remission.

**Table 3:** Effect of oxygen treatment on HRSD sub-scale factors improvement.

HRSD subscale factor	O_2_%	Mean ± SD		Between group effect	
		Baseline	Week 4	Baseline	Week 4
#1—Anxiety/somatization	35% O_2_	3.96 ± 1.5	2.75 ± 1.5	95% CI − 0.95 to 0.78; P = 0.85	95% CI − 2.24 to − 0.51; P = 0.003*
	21% O_2_	4.04 ± 1.4	4.09 ± 1.3		
#2—Weight	35% O_2_	1.2 ± 0.8	0.6 ± 0.7	95% CI − 0.39 to 0.53; P = 0.76	95% CI − 0.87 to − 0.01; P = 0.045
	21% O_2_	1.1 ± 0.7	1.0 ± 0.7		
#3—Cognitive disturbance	35% O_2_	2.5 ± 1.4	1.5 ± 1.3	95% CI − 0.99 to 0.51; P = 0.52	95% CI − 1.66 to − 0.28; P = 0.007*
	21% O_2_	2.8 ± 0.9	2.5 ± 0.9		
#4—Diurnal variation	35% O_2_	0.9 ± 0.7	0.7 ± 0.6	95% CI − 0.74 to 0.74; P = 0.11	95% CI − 0.25 to 0.45; P = 0.56
	21% O_2_	0.7 ± 0.6	0.6 ± 0.5		
#5—Retardation	35% O_2_	4.0 ± 1.1	3.3 ± 1.2	95% CI − 0.63 to 0.79; P = 0.83	95% CI − 1.32 to 0.09; P = 0.086
	21% O_2_	4.0 ± 1.1	3.9 ± 1.1		
#6—Sleep disturbance	35% O_2_	2.8 ± 2.1	1.8 ± 1.7	95% CI − 0.90 to 1.27; P = 0.74	95% CI − 1.53 to 0.46; P = 0.29
	21% O_2_	2.6 ± 1.7	2.4 ± 1.8		

*CI* confidence interval, *HRSD* Hamilton rating scale for depression, *SD* standard deviation.

*P < 0.008 (significant).

### Effects of oxygen treatment on Clinical Global Impression (CGI) score

**Figure 3:**
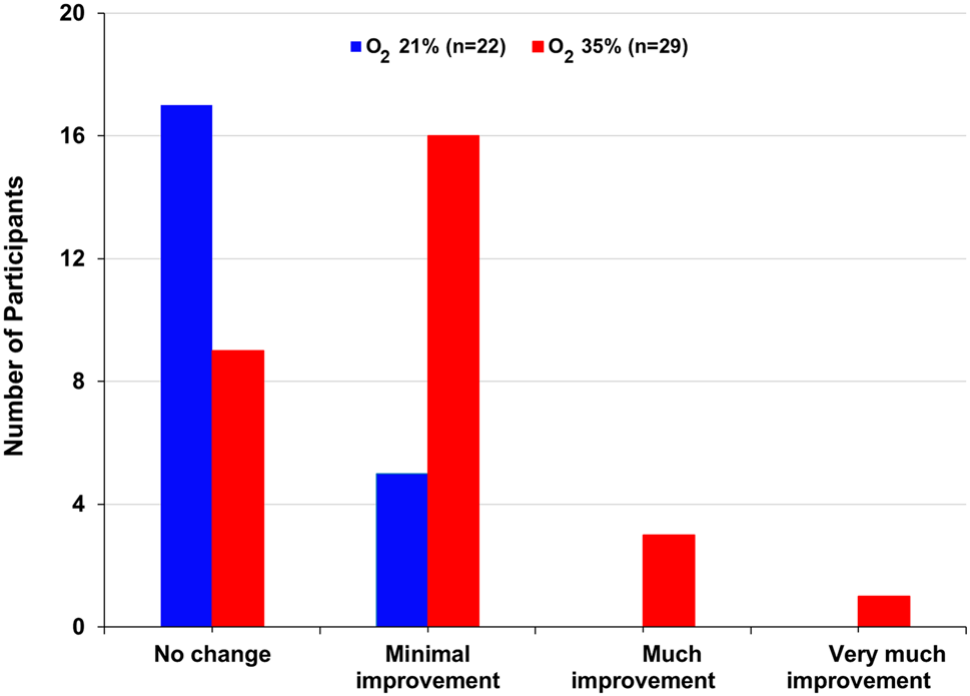
Effect of 35% oxygen treatment on CGI improvement score at 4 weeks. The data are presented as the change from baseline to week four. There were significantly more improved patients in the 35% oxygen treatment group as opposed to the control group, as measured by CGI improvement (Chi-square 11.5, df = 3, P < 0.01).

Table 2 and Fig. 3 show the effect of oxygen treatment on CGI improvement score. Oxygen treatment led to a significant reduction in CGI score compared with room air (mean ± SD, 3.13 ± 0.74 vs. 3.77 ± 0.43) (Table 2). At week four, there was a between-group difference of 0.71 points (95% CI − 1.00 to − 0.29; P = 0.001). The point estimate of the mean treatment effect on CGI score was − 0.078, 95% CI [− 0.313, 0.156]. Of note, the variable “age of onset of depression” did not have a significant effect on CGI-I score (F (18, 42) = 1.66, Prob > F = 0.0888). There were significantly more improved patients in the oxygen treatment as opposed to the control group, as measured by CGI (Fig. 3; Chi-square 11.5, df = 3, P < 0.01).

### Effects of oxygen treatment on the World Health Organization-5 Well-being Index for estimation of Quality of Life (WHO-5-QOL) score

**Figure 4:**
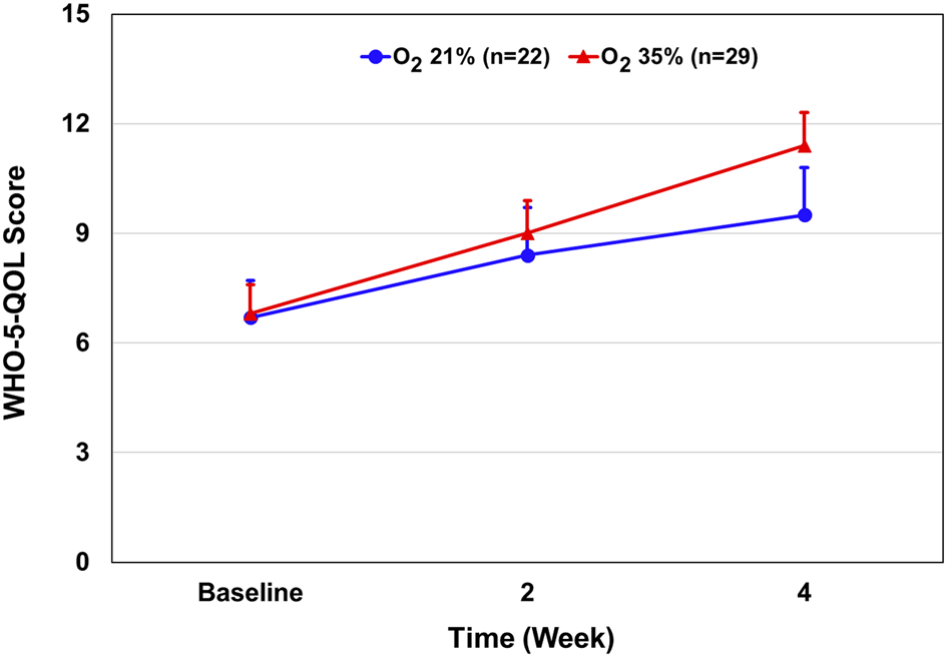
Effect of 35% oxygen treatment on WHO-5QOL score. Results are presented as mean ± SEM. Multivariate regression analysis: The mean ± SD changes in the scores from baseline to week four were 4.5 ± 0.5 points in the oxygen-treated group and 2.6 ± 1.44 in the control group, for a between-group difference of 1.9 points (95% CI − 1.08 to 1.86; P = 0.597).

There was no significant difference in the WHO-5-QOL score between the groups after 4 weeks of oxygen versus room air treatment (mean ± SD, 11.4 ± 4.85 vs. 9.5 ± 6.1) (Table 2). The mean ± SD changes in the scores from baseline to week four were 4.5 ± 0.5 points in the oxygen-treated group and 2.6 ± 1.44 in the control group, for a between-group difference of 1.9 points (95% CI − 1.08 to 1.86; P = 0.597) (Fig. 4).

### Effects of oxygen treatment on the Sense of Coherence (SOC)-13 score

**Figure 5:**
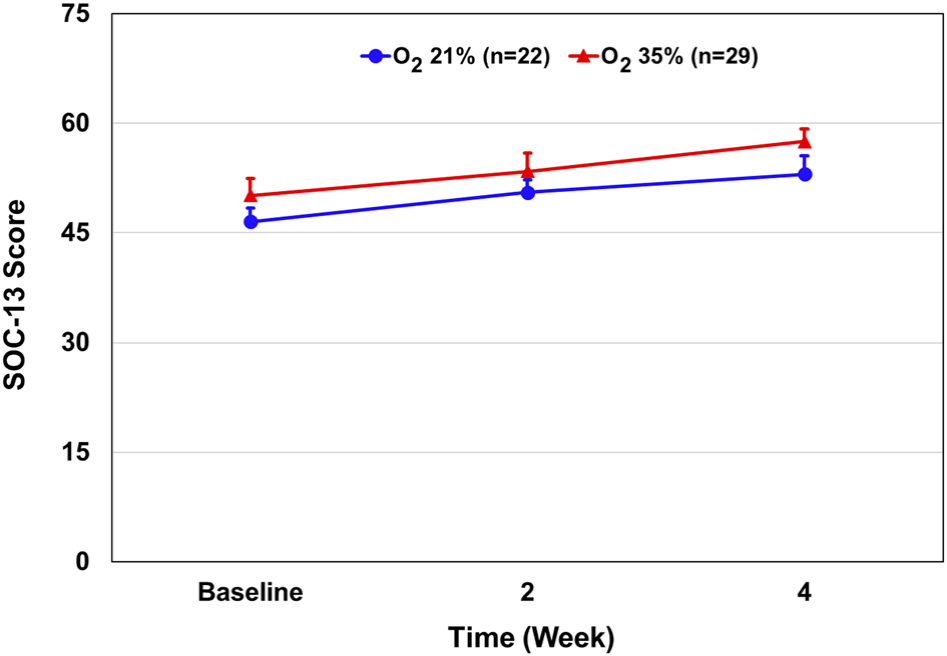
Effect of 35% oxygen treatment on SOC-13 score. Results are presented as mean ± SEM. Multivariate regression analysis: The mean ± SD changes in the scores from baseline to week four were 7.2 ± 3.7 points in the oxygen-treated group and 6.2 ± 2.9 in the control group, for a between-group difference of 0.97 points (95% CI − 3.18 to 9.99; P = 0.303).

There was no significant difference in SOC-13 score between the groups after 4 weeks of oxygen versus room air treatment (mean ± SD, 57.3 ± 9.16 vs. 53.0 ± 11.73) (Table 2). The mean ± SD changes in the scores from baseline to week four were 7.2 ± 3.7 points in the oxygen-treated group and 6.2 ± 2.9 in the control group, for a between-group difference of 0.97 points (95% CI − 3.18 to 9.99; P = 0.303) (Fig. 5).

### Effects of oxygen treatment on Sheehan Disability Scale (SDS) score

**Figure 6:**
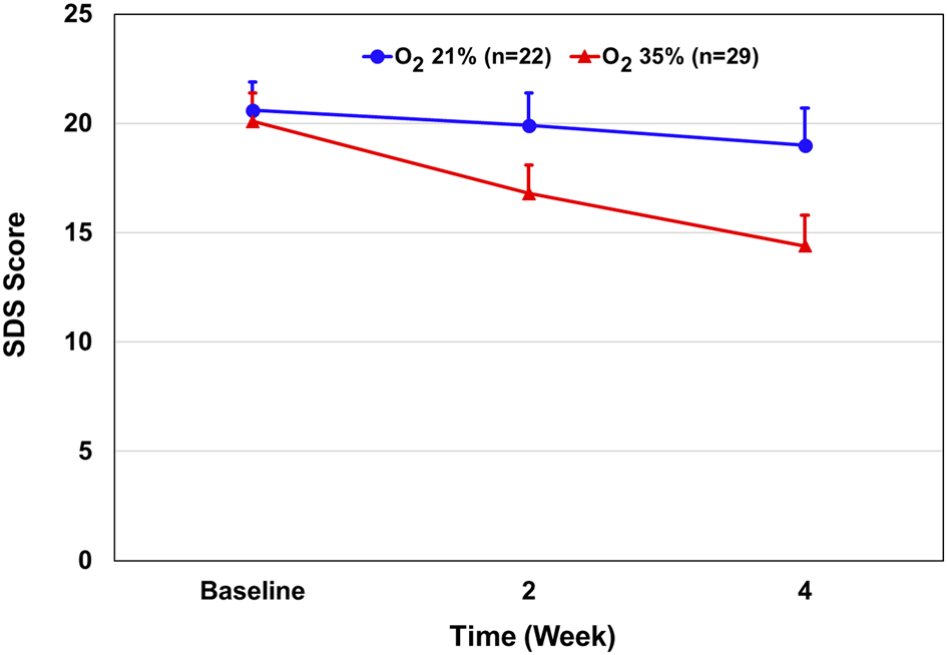
Effect of 35% oxygen treatment on SDS score. Results are presented as mean ± SEM. Multivariate regression analysis: The mean ± SD changes in the scores from baseline to week four were − 5.7 ± 0.6 points in the oxygen-treated group and 1.6 ± 2.2 in the control group, for a between-group difference of 4.1 points (95% CI − 9.84 to 0.22; P = 0.06).

There was no significant difference in SDS score between the groups after 4 weeks of oxygen versus room air treatment (mean ± SD, 14.4 ± 7.6 vs. 19.0 ± 8.0) (Table 2). The mean ± SD changes in the scores from baseline to week four were − 5.7 ± 0.6 points in the oxygen-treated group and 1.6 ± 2.2 in the control group, for a between-group difference of 4.1 points (95% CI − 9.84 to 0.22; P = 0.06) (Fig. 6). Thus, oxygen treatment trended a positive although non-significant effect on coping ability among oxygen-treated subjects.

### Tolerability

Side effects were assessed in all participants at 2 and 4 weeks after treatment initiation. The double-blind treatment was well tolerated in both groups. Four participants dropped-out of the study: one oxygen-treated and three air-treated. Reasons for drop-out: One participant was not interested in continuing without giving a particular reason; two participants were bothered by the noise arriving from the oxygen generator; and, one participant complained of dizziness. Thus, only one patient reported an adverse event—dizziness—which he associated to the treatment he received. To note, this patient was treated with the placebo (room air).

## Discussion

The present study examined the effect of normobaric oxygen treatment (~ 35% O_2_) on symptoms of subjects with depression. Oxygen treatment resulted in a significant reduction in HRSD total score and benefit in the CGI score, without causing significant side effects. These findings deserve replication and future study. The severity of the symptoms of depression was examined using the HRSD scale. Results showed that oxygen therapy significantly reduced the overall HRSD value by four points in 29 patients after 1 month of treatment (Table 2 and Fig. 2). There was a difference of 3.5 points in HRSD between the two groups. A multivariate regression analysis of the HRSD sub-scale factors revealed that the improvement in HRSD score in oxygen-treated subjects was mainly due to a significant reduction in *factor 1*—anxiety/somatization (P = 0.003) and factor 3—cognitive disturbance (P = 0.007), compared with the absence of significant effects on the other parts of the HRSD factors (Table 3). The effect of oxygen treatment on CGI score was also examined. At 4 weeks, the CGI score was significantly lower (P = 0.001) in the oxygen-treated group as compared to the control group, with 0.7 points difference between the two groups (Table 2). Consistently, 69% of the patients in the oxygen treatment group improved compared with ~ 23% in the control group (Fig. 3). Moreover, oxygen treatment non-significantly (P = 0.06) attenuated the disability score as measured by SDS (Table 2 and Fig. 4), suggesting that it may improve the coping ability of participants. In contrast, oxygen treatment did not improve the general well-being (WHO-5-QOL) and did not increase the SOC score of treated subjects.

The positive effects of oxygen therapy on depression symptoms were achieved within 1 month of treatment. A significant difference between the oxygen treatment group and the control group was demonstrable only after four continues weeks of oxygen therapy. It is possible that a more significant improvement could be achieved if oxygen treatment were to be administered for a longer period of time. Thus, future studies should examine whether a longer treatment duration results in a more significant oxygen treatment effect. Moreover, the present study included outpatients of mild to moderate depression severity. It would be interesting to test the causatum of oxygen treatment among different levels of depression severity, including in-hospital patients with severe depression. It should also be studied with different oxygen doses (FiO_2_). The oxygen fraction could perhaps be increased up to 45% without incurring significant safety risks^29,42^. In this regard, the currently available data do not enable drawing unequivocal conclusions regarding the long-term safety of normobaric oxygen treatment among non-hypoxic subjects^43^. Early studies revealed that moderate normobaric oxygen treatment (30–40% O_2_) was safe and did not cause adverse effects or signs of toxicity^23^. However, recent studies demonstrated conflicting results. For example, Stub et al*.*^44^ reported that normobaric oxygen treatment (oxygen via face mask at 8 L/min) in non-hypoxic patients with ST-segment elevation myocardial infarction was associated with increased early myocardial injury and larger infarct size at 6 months post-myocardial infarction. A comprehensive meta-analysis study revealed that liberal administration of oxygen in non-hypoxic acutely ill patients was associated with significantly increased mortality^45^. However, this meta-analysis included many trials in which the oxygen treatment was given at a relatively high fraction of inspired oxygen (FiO_2_ 80–100%). In contrast, Hofmann et al*.*^46^ tested the efficacy and safety of normobaric oxygen treatment (oxygen via face mask at 6 L/min) in non-hypoxic patients with acute myocardial infarction. They found that the treatment was not associated with harmful effects, including absence of increased early myocardial injury. Moreover, a large-scale randomized clinical trial in acute stroke patients showed that oxygen treatment (FiO_2_ 30%) was not associated with any significant adverse effects^30^. This study also showed that nocturnal oxygen treatment (10 h per night) was associated with better safety outcomes as compared to continuous (24 h per day) oxygen treatment. This is pertinent to the design of the present study because we also administered oxygen treatment only during the night, for approximately 7 h per night. Other studies showed that even oxygen treatment of a high fraction of inspired oxygen is safe and possibly effective in severely ill patients^42,47,48^.

As to the tolerability of oxygen treatment, the present study demonstrated that it is safe and did not lead to side effects. Two patients dropped out of the study due to the noise produced by the oxygen generator. If oxygen therapy becomes more common among patients with depression, the problem of noise from the oxygen device can easily be overcome by connecting patients to an oxygen container as a source of oxygen, similar to the one used for respiratory problems. In the present study, patients received a 7.5-m long nasal-tube. For most of them, this allowed for placement of the machine at a far enough distance so as not to be disturbed by the noise. There were also patients who conversely mentioned that the machine's noise helped them sleep. The oxygen generator used in this study weighed 24.5 kg. Previous studies reported that some patients perceived a decrease in quality of life due to their described experience of oxygen generators being heavy and cumbersome^49^. Today this obstacle can be easily defeated as there are smaller units, even portable. Although more expensive, smaller units may facilitate the transportation to and from the patient's home. Some subjects reported that they would willingly pay for portable oxygen systems out-of-pocket because of the mobility it provides^49^. Portable oxygen devices simplify and impel patient therapy; subjects preferred using a portable oxygen systems both at home and during ambulation^50^.

The present study did not explore the biological mechanism underpinning the beneficial effects of *normobaric* oxygen treatment in subject with depression. As mentioned, improvement of mitochondrial function^19^ may contribute to therapeutic effects of normobaric hyperoxia. Normobaric hyperoxia has been reported to influence the function of numerous cellular pathways in the brain, including oxygen sensing mechanisms, pro-survival pathways such as protein kinase B (Akt), mitogen-activated protein kinases, neurotrophins such as brain-derived neurotrophic factor, erythropoietin and its receptors, neuroglobin, nitric oxide, and carbon monoxide^22^. Albeit through different mechanisms, studies in patients with post-stroke depression have shown that *hyperbaric* oxygen treatment reduces the severity of depression symptoms^51,52^. In animals, hyperbaric oxygen treatment was associated with beneficial neuroprotective effects following brain insults such as traumatic brain injury^53^ and focal cerebral ischemia^54^. These studies revealed that hyperbaric oxygen treatment exerts anti-neuroinflammatory effects including an increment of brain levels of the anti-inflammatory cytokine interleukin (IL)-10^53^ and a reduction in brain levels of the pro-inflammatory cytokines IL-1β and tumor necrosis factor-α, and, a decrease in myeloperoxidase activity^54^.

Our statistical analyses clearly demonstrated that the positive therapeutic effects of oxygen treatment did not derive from differences in the rate of adherence to treatment between the groups. Namely, the multivariate regression analysis revealed that the variable "adherence to treatment" (as measured by average exposure time to the treatment regimens) did not have a significant influence on the effect of oxygen treatment on HRSD score (95% CI − 0.0695 to 0.0056; P = 0.093). Similarly, all other tested outcomes (CGI, SDS, WHO-5-QOL, and SOC-13 scores) were not affected by adherence to treatment. Moreover, the positive effects were not due to differences in basic or clinical characteristics of participants. Interestingly, current antidepressant drugs do not have a mood elevating effect in normal persons, whereas drugs like amphetamines do. We speculate that the oxygen treatment exerts an antidepressant effect rather than a mood-elevating effect. A study of normal volunteers given normobaric oxygen treatment has not yet been done and would be an important study to do in the future. “Oxygen bars” have become popular in Japan and other East Asian countries, said to generate mood elevating effects^55^. In an effort to unveil this unknown, normal volunteers might be studied with normobaric oxygen treatment administered during a night sleep and consequently exploring a possible elevation in mood the following day.

One can compare the reduction in HRSD with oxygen treatment in the present study to that of patients with depression in two previous studies in the Beer-Sheva, Israel population^56,57^. One study^56^ of inositol treatment of depression was a double-blind trial; 13 patients were given inositol and 15 patients were given placebo. Over 4 weeks of treatment the HRSD score declined significantly more for those inositol treated than for the placebo. The mean reduction of HRSD in the patients receiving inositol was 10.8 points, compared to four points in the placebo group, a nearly seven-point difference. In another study^57^, omega-3 fatty acids treatment was given to a mixed group of patients, some taking antidepressants and some not, as in the present study. The mean reduction of HRSD score in the patients receiving omega-3 fatty acids was 12.4 points, compared to 2.3 points in the placebo group, culminating in roughly a 10-point difference. In this regard, it is worth noting that recent meta-analysis studies have demonstrated much smaller effect sizes of omega-3 fatty acids^58,59^ as well as classic antidepressant drugs^60,61^ as a treatment for depression. In our study, the decline in HRSD over the course of 4 weeks was 4.2 points in the oxygen group and 0.7 points in the control group, about a 3.5-point difference. It is widely reported that the placebo-treatment difference in depression studies has markedly declined worldwide over the last 30 years^62–64^. Moreover, the Levine et al*.*^56^ and Nemets et al*.*^57^ studies recruited patients from an outpatient psychiatry clinic, whereas the current study recruited participants from family medicine clinics.

### Limitations

The present study has several limitations. First, it included a relatively small sample size. However, a small sample is acceptable in pilot studies that investigate novel therapeutic interventions in psychiatric patients^65–67^. Second, this study did not explore the biological mechanism underlying the therapeutic effects of normobaric oxygen treatment on the brain. Third, we did not follow the patients after the last planned assessment (4 weeks), so we cannot speculate whether the observed relief in depressive symptoms is durable. Fourth, the external validity of the results is limited because the study was conducted in a particular group of patients; outpatients with mild to moderate depression who live in the southern district of Israel.

## Conclusions

This pioneer, proof-of-concept, double-blind study showed that normobaric oxygen treatment is beneficial in patients with depression. These results require further examination and replication in future studies of larger cohorts. Given that normobaric oxygen treatment is a simple, not invasive and safe therapeutic intervention makes it a potential and attractive treatment for depression.

## Methods

All methods in the study were carried out in accordance with relevant guidelines and regulations.

### Study design and participants

This randomized, double-blind study lasted 4 weeks. Participants were recruited to the study through two routes: (1) subjects who directly contacted the investigators expressing interest in participating in the study following advertisement in public media; and, (2) subjects from *Clalit Health Services* in Beer-Sheva, Israel, who were referred to the study by their family physicians. An initial screening for depression was done by an experienced psychiatric nurse, using a questionnaire based on the Structured Clinical Interview for Depression^68^. Patients deemed suitable were interviewed by a board-certified psychiatrist in order to confirm *Diagnostic and Statistical Manual of Mental Disorders-4* depression^69^ and approve participation in the study. Only participants who had a HRSD score ≥ 8 and provided written informed consent to participate in the study were included. The cut-off of ≥ 8 for the HRSD was based on data from previous studies^39,70^. *Inclusion Criteria*: Men and women aged 18–65 years with mild to moderate depression (HRSD score 8–18)^39,70^; and, blood oxygen saturation ≥ 95% at room air. Blood oxygen saturation at baseline was assessed using a high precision *Pulse Oximeter* (*CONTEC Medical Systems Co., Ltd.*; Shanghai, China). *Exclusion Criteria*: An unstable psychiatric condition; a need for change in current psychiatric medication prescriptions; suicidal ideations or suicide attempts at the time of recruitment; drug abuse; acute or chronic respiratory disease; any severe physical illness; obesity (body mass index [calculated as weight in kilograms divided by height in meters squared] > 30); non-Hebrew speakers; or inability to cease smoking during night hours while participants were supposed to use the oxygen supplementing machine. Four hundred and ten subjects were assessed for suitability and underwent an initial screening (Fig. 1). Fifty five participants met the inclusion criteria of the study, signed an informed consent form to participate and underwent randomization (Fig. 1).

### Sample size

During the planning phase of the study, we assumed that most of the participants we would recruit would already be on pharmaceutical antidepressant treatment (this estimate turned out to be correct; 60% of the study participants were on antidepressant treatment at baseline, Table 1), thus, the addition of oxygen/placebo treatment would serve as an “add-on” therapy. Published studies of add-on therapies in depressive patients have shown that there is a chance of gaining an additional 30 to 50% response in the add-on treatment groups^71,72^. Based on these findings, we performed a power analysis using the following parameters: Two-sided significance level = 95%; power = 80%; desired ratio between the groups 1:1. The calculation revealed that we should recruit 40 subjects to each group (80 subjects in total for the trial). However, after initiating the trial, we came to know that it was very difficult to recruit participants to a study investigating a novel treatment strategy wherein the utility and tolerability of which are entirely unknown to enrollees. After 6 years of enrollment (2014–2019) and recruitment of 55 subjects, we consulted with our advising committee and jointly decided to discontinue recruitment. Our decision was based on two considerations: (1) The prolonged trial duration may influence the outcomes of the trial (time-effect), and, (2) a relatively small sample is acceptable in randomized clinical trials that investigate novel therapeutic interventions in psychiatric patients^65–67^.

### Procedure and randomization

An oxygen generator is a portable unit that was easily translocated to the home bedside of participants. The machine supplies highly oxygen-enriched air (up to 99% O_2_). By setting a flow rate of 5–6 L/min^73^, 35% oxygen is delivered to the lungs when nasal tubes are used (as in our study). Machines utilized in the control and intervention groups looked and sounded identical; it was not possible for field-researchers or participants to know which treatment each participant received. Machines used as controls were inactivated by disconnection of an internal pipeline that transfers oxygen, thereby impeding on the technological mechanism and resulting in air-crossing. The procedure was performed by a certified medical equipment laboratory of *Clalit Health Services* under the direction of the investigator who was in charge of generating the allocation sequence (Yu.Be.). This investigator was aware of the particular settings (treatment) of each machine and, therefore, was *not* involved or present in any assessment of study participants. He provided the coded machines to the field-researcher (Ye.Bl.) and she handed them to participants blinded to the treatment allocation. The safety and technical compatibility of the oxygen generators were monitored by a designated manufacturer’s technician. The safety and compatibility evaluations of the machines were conducted after completion of every treatment course (up to 1 month treatment for individual patients) and included the following: assessment of machine completeness and general safety (e.g., power cord), cleaning of machine filters, inspection for air/oxygen leaks, assurance of accurate oxygen supply (flow and FiO_2_). Patients were instructed not to smoke near the device. Participants were monitored by the research nurse who oversaw their safety and adherence and executed the rating scale assessments at designated time points. Subjects received a 7.5-m long oxygen tube allowing the machine to be placed at a convenient distance from their bed.

### Study intervention

Consecutive participants were randomly assigned to the study groups according to an allocation sequence designated to generate a balanced ratio between the groups. Subjects in the intervention group were given treatment with oxygen-enriched air (35% O_2_) through a nasal tube, 7 h per night for 1 month. Subjects in the control group were given treatment with room air (21% O_2_) through a nasal tube, 7 h per night for 1 month. It is important to reiterate that the procedure of supplying 35% O_2_ or 21% O_2_ to study participants appeared identical in its entirety. Neither the research nurse nor the participants could recognize which FiO_2_ each participant received; namely, both the research nurse and the participants were blind to the treatment allocation.

### Assessment tools

(1) *The 21-item HRSD*^39,70,74^—This is a clinician rated scale aimed at assessing depression severity. It includes 21 items intended to assess: depressed mood; feelings of guilt; suicide; insomnia; work and activities; retardation; agitation; anxiety (psychological and somatic); somatic symptoms (gastrointestinal and general); genital symptoms; hypochondriasis; weight loss; insight; diurnal variation; derealization/depersonalization; paranoid symptoms; and obsessive/compulsive symptoms^39,70,74^. Of the 21 items, ten have scores ranging from 0 to 4 points, nine with scores ranging from 0 to 2 points, and two with scores ranging from 0 to 3 points. Thus, the general score may be between 0–64. The widely accepted cut-off scores are: < 8, normal; 8–13, mild depression; 14–18, moderate depression; 19–22, severe depression; > 23, very severe depression^40,41,45^. The inter-rater reliability of the 21-item HRSD was found high (correlation coefficient = 0.81) in a randomized, placebo-controlled, double-blind trial of patients with treatment-resistant depression^41^. (2) *The CGI scale*^75^—This scale comprises two companion one-item measures evaluating the following: (i) Severity of psychopathology (on a scale of 1–7) and (ii) change from the commencement of treatment (on a scale of 1–7). CGI-severity is rated on the following scale: 1 = normal, not at all ill; 2 = borderline mentally ill; 3 = mildly ill; 4 = moderately ill; 5 = markedly ill; 6 = severely ill; 7 = among the most extremely ill patients. CGI-improvement (change): 1 = very much improved since initiation of treatment; 2 = much improved; 3 = minimally improved; 4 = no change from baseline; 5 = minimally worse; 6 = much worse; 7 = very much worse since initiation of treatment^75^. (3) *WHO-5-QOL*^76^—This is a five-item instrument designed to measure overall quality of life. Each of the five items is rated on a 6-point scale: 0 = not present; 5 = constantly present. The theoretical score ranges from 0 to 25. Higher scores mean better well-being^76^. A score below 13 indicates poor well-being and is an indication for testing for depression. (4) *SOC* 13-items questionnaire^77^—The SOC theory is a theoretical model that explains successful coping with stressors^78^. According to the theory, the stronger the SOC, the more likely the person will be able to cope with life stressors. The questionnaire includes 13-items on a 7-point scale, with two anchoring responses: “never” and “very often”. The range of the score is between 13 and 91; a high score represents a strong SOC^77,79^. The instrument has a Cronbach´s alpha of 0.74–0.91 and r = 0.91^79^. (5) *SDS*^80^—This instrument includes three self-rated items designed to measure the extent to which (i) work, (ii) social life/leisure activities, and, (iii) home life or family responsibilities, are impaired by panic, anxiety, phobic or depressive symptoms^80^. Each item is rated on a 0–10 scale. The three items may be summed into a single dimensional measure of global functional impairment that ranges from 0 (unimpaired) to 30 (highly impaired). The tool has a Cronbach’s alpha of 0.89^81^. Using these tools, assessments were performed at three time points: baseline, 2 weeks and 4 weeks after treatment initiation. All clinical ratings were performed in a family medicine clinic of *Clalit Health Services* under the direction of one of the authors (P.S.). The nurse who performed all assessments (Ye.Bl.) was blinded to the treatment conditions of each participant.

### Adherence to treatment

Participants of the study were administered oxygen-enriched air or room air for 7 h per night for a month. The rate of adherence to treatment was assessed by measuring the amount of exposure time to treatment regimens/time using the oxygen generator by participants. The maximal possible amount of treatment hours for each participant during the study was 210 h (7 h × 30 days). There was no significant difference between the oxygen-treated group and the room-air-treated group in the number of hours using the oxygen generator (T-test; mean ± SD, 120.1 ± 29.62 vs. 124.6 ± 33.97, df = 49, t = 0.506, 95% CI − 22.5 to 13.4, P = 0.62) nor in the proportion of participants with moderate or high rate of adherence (Chi-Square test; P = 0.08).

### Statistical analyses

A multivariate regression analysis was performed using *Stata 12.0* (Stata Corp, College Station, TX, USA), according to accepted methodology^82^. The *Lagrange Multiplier Test*^82^ examines the effect on the first order conditions for a maximum of the likelihood of imposing parametric hypothesis. There have been several reports illustrating and advocating the use of this method for multivariate analysis, particularly, multivariate regression. The key property of multivariate regression using the Lagrange Multiplier test is that several dependent variables are jointly regressed on the same independent variables. In the present study, the analysis determined the difference between the groups by comparing the mean and standard error of mean of the groups in terms of HRSD, CGI, SOC-13, WHO-5-QOL and SDS values. In our analysis of total HRSD score, the reported significance level for oxygen treatment is 0.007, which approximately equals to 0.05/8 (where eight is the number of the independent variables in the equation). A multivariate regression analysis was also preformed stratifying each subscale factor of the HRSD to the total change from baseline to week four. In this analysis, the reported significance level for each factor is 0.008, which approximately equals to 0.05/6 (where six is the number of the sub-scale factors of the HRSD). Calculation of the effect of oxygen treatment and contribution of each subscale factor of the HRSD to the total change from baseline to week four was done as follows: Δ value of subscale factor (baseline − week four)/Δ total HRSD score (baseline − week four) × 100. Differences in basic and clinical characteristics between the groups were tested using *T*-test (parametric variables) and chi-square test (non-parametric variables). Chi-square test was used and Yates correction was performed in cases with less than 10 points in a cell. Values of P < 0.05 were considered statistically significant.

### Ethical considerations

Only participants who provided written informed consent to participate in the study were included. This study was approved by the institutional ethical review board (Helsinki Committee) of *Clalit Health Services* (approval # 051/2013). The trial registration number at *NIH.GOV* was NCT02149563 (29/05/2014). The study was funded by the *National Alliance for Research on Schizophrenia & Depression* Independent Investigator Award (to Yu.Be.).

## Data Availability

The data that support the findings of this study are available from the corresponding author upon reasonable request.
